# Supplementary material for: Virtual monochromatic imaging with projection-based material decomposition algorithm for metal artifacts reduction in photon-counting detector computed tomography
Source: PLoS One. 2023 Mar 13;18(3):e0282900. doi: 10.1371/journal.pone.0282900 (PMC10010526; doi:10.1371/journal.pone.0282900)
Supplement: Attachment — Submitted filename: Response to Reviewers_submit.pdf [file pone.0282900.s001.pdf]

We would like to thank the referees for their insightful comments that have helped us clarify and strengthen the manuscript. We have addressed the requests of the referees and provided below point-by-point detailed responses to the questions. A version of the manuscript with all corrections highlighted is submitted along with a revised clean version of the manuscript as requested.

Reviewer #1:

1. We need to improve the English grammar of overall text in the paper.
   [Response] Thanks for the reviewer's suggestion. The manuscript has been edited by a native English speaker.

2. The overall introduction and history of the research is impressive, almost like review paper. However, the importance of projection domain material decomposition is somewhat out of the ordinary.
   [Response] We appreciate this concern and have added more description of projection domain material decomposition in Line 120-131.
   …"Compared to image-based MD, the projection-based MD method offers more accurate image quantification of contrast agents and fewer artifacts caused by beam hardening[26]." …. "Despite image-based MD being computationally faster, projection-based MD provides better MD results [26]. Furthermore, projection-based MD is the more precise model that directly decomposes projection data rather than after the procedure of reconstruction [30]. Hence, projection-based MD is more suitable to generate VMI with the ability of metal artifact reduction in the CT images."

3. Parameters of the developed CT
   - The FOV of the developed machine seems a bit small.
   [Response] For commercial PCD products, the extension of the active detection area has limitations due to the design of the analog front-end readout circuit and the cost of the detector manufacture [Ref. 1-1]. Hence, most PCD assemblies are in linear or small area type [Ref. 1-2]. The simple specifications comparison of PCD and EID is listed in Table R1-1. The active detection area of PCD is much smaller than EID. The price per unit area of PCD is 36 times higher than EID. For previous studies of PCD-CT, a small area PCD (~155 × 13 mm^2) is also used to perform spectral micro-CT experiments [Ref. 1-3, Ref. 1-4]. Hence, the small area PCD was used to construct a spectral micro-CT prototype system to reduce the cost of system assembly. We acknowledged that the FOV of our developed PCD-CT is small, but we consider it a versatile CT demonstrator that helps us to further develop advanced technology for PCD-CT. In addition, we believe that the price of PCD will be reduced with the detector technology development.

Table R1-1. The simple specification comparison of PCD and EID.

Detector Type	EID (Energy Integrating Detector)	PCD (Photon Counting Detector)
Manufacturer / Type	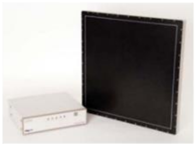 Varex / 4343CB	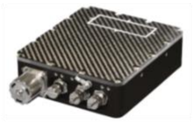 Direct Conversion / XC-Thor
Num. of energy thresholds	N/A	2
Pixel number [pixel]	3,072 × 3,072	1,024 × 512
Pixel size [μm]	139	100
Active detection area [mm × mm]	427 × 427	102.4 × 51.2
Price per unit area [USD/mm^2]	0.5	18

Reference:

[Ref. 1-1] Taguchi K, Iwanczyk J S. Vision 20/20: Single photon counting x-ray detectors in medical imaging. Med Phys. 2013;40: 100901. doi:10.1118/1.4820371

[Ref. 1-2] Direct Conversion website: https://directconversion.com/products/#xcounter

[Ref. 1-3] Wu D, Zhang L, Zhu X, Xu X, Wang S. A weighted polynomial based material decomposition method for spectral x-ray CT imaging. Phys Med Biol. 2016;61: 3749–3783. doi:10.1088/0031-9155/61/10/3749

[Ref. 1-4] Sellerer T, Ehn S, Mechlem K, Duda M, Epple M, Noël P B, et al. Quantitative dual-energy micro-CT with a photon-counting detector for material science and non-destructive testing. PLoS One. 2019;14: 1–18. doi:10.1371/journal.pone.0219659

- Why did you put PCD threshold at 30 to 80 KeV when it was 140kvp? The spectrum range is wider than that, and even with a filter, the photons in 30 under/80 upper spectrum range have inneglectable amount in a scope of beam hardening.

[Response] We apologized for the imprecise description regarding the description of energy thresholds and have added an additional description in Line 316-318.

"The low-energy and high-energy threshold was set at 30 keV and 80 keV, resulting in two energy bins of [31 80] and [81 140] keV."

For the photon energy smaller than 30 keV, the lower energy threshold (30 keV) was used to eliminate image noises induced by the detector’s dark current.

- There will be insufficient areas during the rebinning process with 360 degree bin. Based on the Nyquist frequency, considering the beam angle in the design, it is determined that the sampling rate of the periphery except the center is not sufficient. Considering that 1080 view was used in the numerical simulation, it is expected that the issue is clearly understood from the writer.

[Response] We appreciate this concern and have added the following text to the Results section in Line 553-555.

"Fig 10 reveals view-aliasing artifacts due to insufficient projections within one rotation, but further collect more projections can minimize the effect."

4. The windowing and image resolution of the Figure image are not sufficient to recognize the strike/beam-hardening artifact in the image. Additional work would be needed, such as narrowing windows width of the image.

   [Response] Thanks to this suggestion, we have narrowed the window width of the CT images from 2,000 HU to 1,000 HU (please see Fig 8 and Fig 9). After evaluation of the uploaded image, it appears that the reduced image quality in the manuscript is caused by the PDF generation process by PLOS ONE. However, up to our previous experience, the manuscript's image quality would be much improved when it is published.

5. In the linear summing/ray tracing method, how did you design the hardening/scatter that occurs as the beam is transmitted? Just simple integration of the path, or scatting effects were adopted? Does each mass/linear attenuation coefficient values were referred from NIST database? or measured from the developed machine?

   [Response] We appreciate this concern. In this work, we only considered simple integration of the path (without consideration of scatter effect) using the ASTRA-toolbox package. The linear attenuation coefficients for materials of XCAT phantom were referred from the NIST database (so-called NIST-XCOM) [38]. For clarity, the related sentences were added and modified in the sections "Digital XCAT phantoms" and "The simulation setup for PCD-CT imaging", respectively.

6. Through the calibration/test, what is the material range that can be reviewed during the calibration process? Is it possible to cover all the material ranges that can be used in the actual use-case? We all know that models such as TASMIP/MASMIP/SPEKTR have limitations in mimicking the real world data.

   [Response] In response to the concern, we have conducted additional simulation experiments, and the detailed responses are as follows:

   For material decomposition in medical applications, PMMA and Al were used as the basis materials for representing soft tissue and bone, respectively. Besides the use of Titanium (Ti) as the material of metal implants, we performed additional simulation experiments by adding

Copper (Cu) metal implants in the head case of the XCAT phantom for evaluation. Fig R1-1 shows generated VMCT for PCD-CT images of the phantom with Ti and Cu implants based on the PMMA and Al basis materials. Fig R1-1 (a) is the VMI-Atable images the same as the result of Fig 8 in the manuscript. Fig R1-1 (b) shows the generated VMI-Atable images of XCAT phantom with Cu implants under the same thickness range of the basis materials; the metal artifacts are obvious in the VMI-Atable images of the head phantom. However, when the thickness range of Al (basis material) is increased from 10 cm to 15 cm, the metal artifacts of the head VMI-Atable image can be significantly reduced as shown in Fig R1-1 (c).

For metal implants with various materials, the implant material can be decomposed into an effective thickness of the basis materials, even if the implant materials are not the same as the basis materials. Hence, for implants with high attenuation, one can obtain the correct effective thickness of the basis materials in the material decomposition process by increasing the thickness of the basis materials as demonstrated in Fig R1-1 (b) and (c). However, for the extreme scenario such as the use of high-Z materials as metal implants (e.g., Au, Pb …), the requirement of the basis materials thicknesses would increase rapidly. This would lead to basis materials being too thick and increase the difficulty of executing the material decomposition calibration process. Hence, we suggest considering other materials with higher attenuation such as Ti, and Cu as the basis material. In summary, it's difficult to cover all the material ranges in the actual use-case only using only one pair of PMMA-Al as the basis materials. For covering all material ranges, establishing LUTs of multiple basis materials may be a better solution, which merits further investigation.

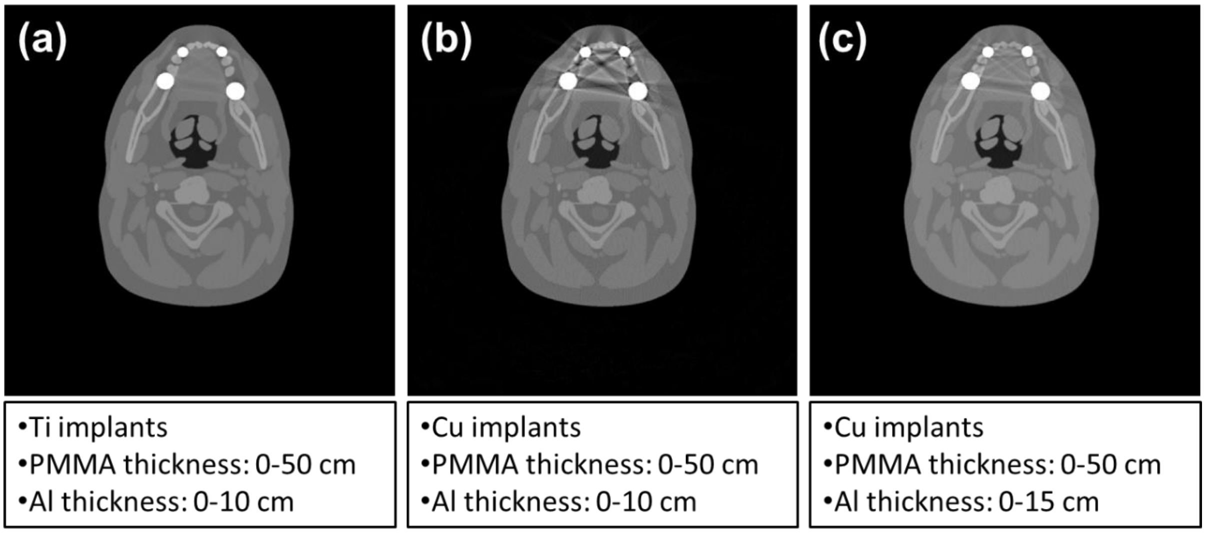

Fig R1-1. Simulated results using the head XCAT phantom for evaluating the two different implant materials.

7. It seems that unification or definition of the expression of the unit would be needed. (Kev, kv,

kVp) it seems that a few typos of the unit were written.

[Response] Thanks for the reviewer's pointing out our omission. For avoiding confusion, we unified the unit of tube voltage as "kV" in text of the manuscript and the unit of monochromatic photon energy as "keV" (kilo-electron volt) for the settings of energy threshold, photon energy bin, and VMI energy.

8. How come did you used 100 KeV monochromatic energy as a reference of non-metal artifact image on 140 KVP polychromatic image? The average KeV in 140 KVP spectrum doesn't seem as close to 100 KeV in TASMIP spectrum.

   [Response] We appreciate this concern and response as follows. The mean energy of the 140 kV TASMIP spectrum with 2-mm thick Al filtration is about 60.2 keV. Some studies have indicated that generating VMI with higher keV can more effectively reduce the effects of beam hardening and metal artifacts [Ref. 1-5]. According to our preliminary study, 100 keV VMI can provide better MAR performance. Therefore, the reference image using 100 keV monochromatic energy was considered for a direct comparison as well.

   Reference:

[Ref. 1-5] Wellenberg R. H. H., et al. Metal artifact reduction techniques in musculoskeletal CT-imaging. Eur. J. Radiol. 2018;107: 60-69. doi: 10.1016/j.ejrad.2018.08.010

9. It seems that the full description of the abbreviation should be specified. (PSNR, NRMSE, etc) we all know that PSNR is Peak-Signal-To-Ratio, however, it may cause confusion to readers. **[Response]** Thanks for this reviewer's suggestion and we have added the full description of the abbreviation PSNR, NRMSE, and SSIM in the "Image quality evaluation" section.

Reviewer #2: General Comments

Metal artifact reduction (MAR) is a major challenge in computed tomography (CT) to improve image quality and medical diagnosis and treatment. The aim of this study is to develop and implement two projection-based material decomposition (MD) algorithms to generate virtual monochromatic CT (VMCT) images for MAR, and evaluate their effectiveness in comparison with two conventional MAR methods, i.e., LMAR and NMAR. The derivation of the proposed methods, VMI-poly and VMI-Atable, are described in the manuscript. The materials and methods include a simulation study using the 3D XCAT phantom with titanimum (Ti) metal implants, and an experimental study using a spectral micro-CT prototype system equipped with a photon-counting detector (PCD) and a homemade metal artifact evaluation (MAE) phantom. Results from the simulation and experimental studies demonstrate that, in comparison to the conventional MAR methods, the projection-based VMCT images can not only reduce metal artifacts effectively but also simultaneously prevents object blurring at the metal artifact position and image distortion of the metal implants.

The manuscript addresses an important challenge in CT and is a timely study. It contributes greatly to the current research efforts in the development of MAR methods to improve CT image quality that will potentially lead to improvement in clinical diagnosis. In general, the different aspects of the study presented in manuscript are clearly described. The materials and methods used in the study are well designed and appropriate. The proposed projection-based MD algorithms and derivation of the VMCT images are clearly presented. Results from the simulation and experimental studies are well-presented and convincing. However, there are several specific comments and concerns listed below that need to be addressed. With satisfactory responses to the specific comments and concerns, the manuscript is recommended for publication in the PLOS ONE journal.

Specific Comments

1. Although in general both the scientific content and the writing of different sections of the manuscript are good, the organization of the manuscript can be improved. A typical scientific manuscript is usually arranged in sections in the order of introduction, materials and methods, results, discussion and conclusion. In this manuscript, some of the topics and sections are not placed in their logical order and can be re-arranged for easier reading by the readers.

   [Response] We appreciate this suggestion. We have rearranged the topics in the section of Materials and Methods in a more logical flow ("Projection-based material decomposition model" and "VMI algorithm" sections were first introduced, follow by sections related to experimental and simulations setup) for easier reading by the readers.

2. Line 437. Please list the full letters of the acronyms, PSNR, NRME and SSIM.

   [Response] Thanks for this reviewer's suggestion and we have added the full description of the abbreviation PSNR, NRMSE, and SSIM in the "Image quality evaluation" section.

3. Line 440. What do you mean by ‘gray values’?
   [Response] We have added an additional description “(HU values of CT images)” to the text ”gray values” in Line 445.

4. Line 458. Please state what the ‘circle’ is in the sentence.
   [Response] For avoiding confusion, we have removed the term of ‘circle’ and re-written the sentence as “In the experiments, the circularity was measured on the rods of MAE phantom images to evaluate the influence of the different MAR methods on image distortions in the metal artifact regions.” in Line 463-465.

5. Line 466. Instead of ‘0 for highly non-circular shapes’, do you mean ‘larger deviation from 1 for greater non-circular shapes’?
   [Response] Per the reviewer’s suggestion, we have modified the sentence as “The value of circularity lies between 0 and 1, and the value has a larger deviation from 1 for greater non-circular shapes.” in the Line 471-472.

6. Line 476. It would be good to replace ‘the conventional MAR methods and projection-based VMI algorithms for MAR’ by ‘the two conventional MAR methods, LMAR and NMAR, and the 2 projection-based VMI algorithms, VMI-Poly and VMI-Atable, for MAR’ at the beginning of the paragraph.
   [Response] Per the reviewer’s suggestion, we have modified the sentence as “The results of the two conventional MAR methods, LMAR and NMAR, and the two projection-based VMI algorithms, VMI-Poly and VMI-Atable, for MAR with the XCAT phantom are presented in Fig 8. ” in Line 479-482.

7. Line 485. It would be good to introduce the terms VMI-poly and VMI-Atable, in the earlier sections where they were first introduced and described.
   [Response] We appreciate this suggestion and first defined the two terms of VMI-poly and VMI-Atable in the “Projection-based MD algorithms and VMI” section.

8. Line 521. Should the ‘different ROIs’ be more clearly listed as ROI #1, ROI #2 and ROI #3 as shown in Figure 8.
   [Response] Upon the reviewer's suggestion, the sentence was modified as follows: “Entire images and three ROIs (ROI #1, ROI #2, and ROI #3) in the reference images were used for comparison (as shown in Figs 8).” (Seen in Lines 524-525).

9. Line 552 and Figs. 10 (b) – (e). The gray-scale of the image are too saturated to see any variations

within the images of the different rods.

[Response] Per the reviewer's suggestion, we have adjusted the window width/window level in CT images from 15000/6500 HU to 11000/4500 HU to present clearer image details in Fig 10.

10. Lines 672 – 674. The sentence does seem to make sense. Please rewrite it.

    [Response] Upon the reviewer's suggestion, we have rewritten the sentence from ”Hence, the CT image quality can be further improved to increase the abilities for both preoperative and postoperative assessment of metal implants.” to “It may thus help improve the assessment of implants and bones, as well as soft tissue surrounding them, in postoperative follow-up examinations.” in Line 675-677.
